# Supplementary material for: The interactome of intact mitochondria by cross-linking mass spectrometry provides evidence for coexisting respiratory supercomplexes
Source: Mol Cell Proteomics. 2017 Dec 8;17(2):216–32. doi: 10.1074/mcp.RA117.000470 (PMC5795388; doi:10.1074/mcp.RA117.000470)
Supplement: Supplemental Data [file supp_RA117.000470_133922_0_supp_23978_fzffwf.zip › spectra_annotation/mito_spectra_annotation/252-1-4-1-13-1.pdf]

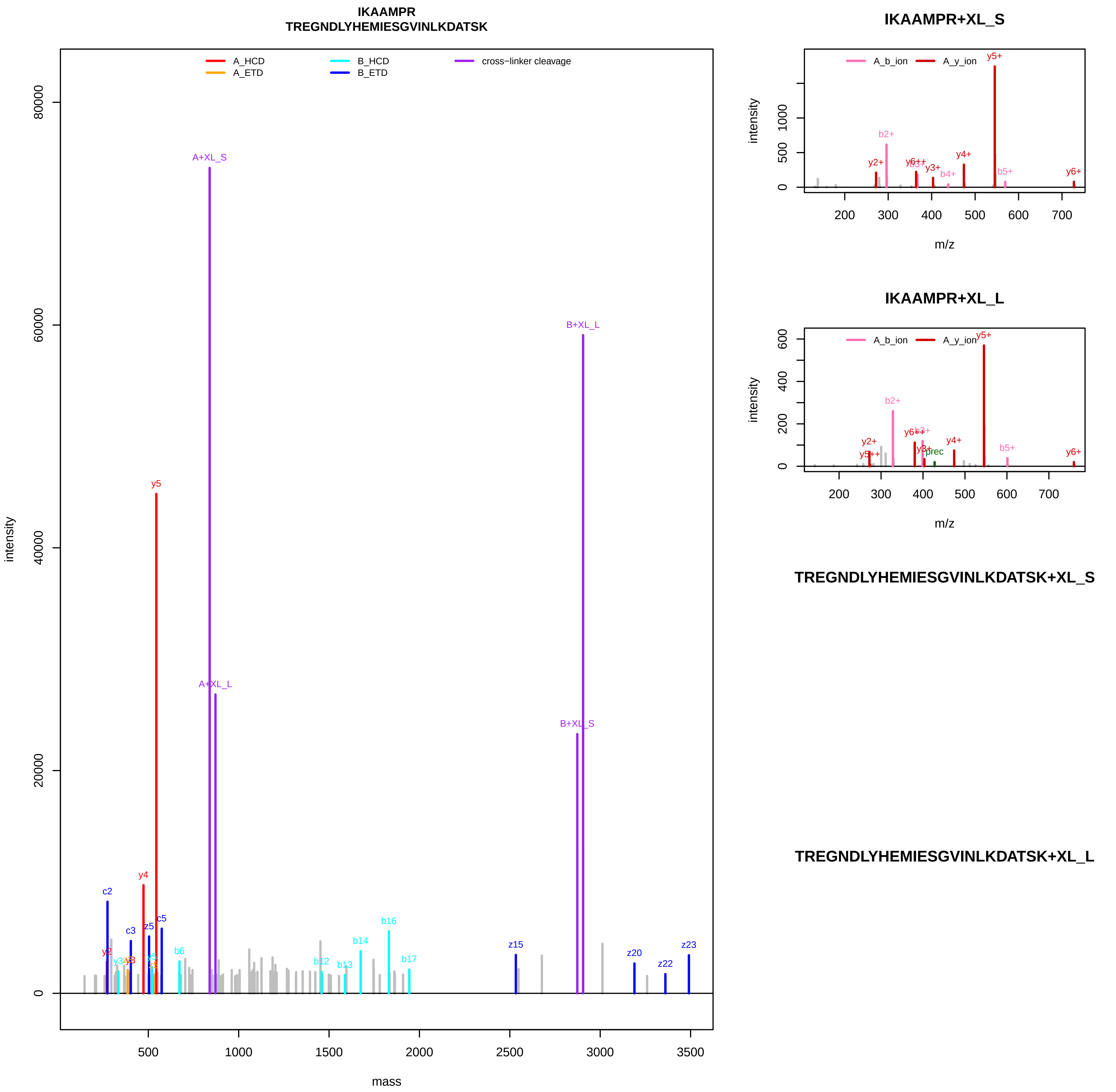
IKAAMPR
TREGNDLYHEMIESGVINLKDATSK
A_HCD
A_ETD
B_HCD
B_ETD
cross−linker cleavage
A+XL_S
A+XL_L
B+XL_L
B+XL_S
y5
y4
y2
y3
c2
c3
c5
z5
b6
b12
b13
b14
b16
b17
z15
z20
z22
z23
intensity
mass
IKAAMPR+XL_S
A_b_ion
A_y_ion
y5+
b2+
y2+
y6++
y3+
b4+
y4+
b5+
y6+
m/z
IKAAMPR+XL_L
y5++
b3+
prec
TREGNDLYHEMIESGVINLKDATSK+XL_S
TREGNDLYHEMIESGVINLKDATSK+XL_L
